# Point-of-care Ultrasound Diagnosis of Bilateral Patellar Tendon Rupture

**DOI:** 10.5811/cpcem.2019.10.44194

**Published:** 2020-01-24

**Authors:** Kathleen Ogle, Sohaib Mandoorah, Matthew Fellin, Hamid Shokoohi, William Probasco, Keith Boniface

**Affiliations:** * George Washington University, Department of Emergency Medicine, Washington, District of Columbia; † George Washington University, Department of Orthopedics, Washington, District of Columbia

## Abstract

Musculoskeletal complaints are one cornerstone of urgent issues for which orthopedic and emergency physicians provide care. Ultrasound can be a useful diagnostic tool to help identify musculoskeletal injuries. We describe a case of bilateral patellar tendon rupture that presented after minor trauma, and had the diagnosis confirmed at the bedside by point-of-care ultrasound. Physicians caring for patients with orthopedic injuries should be familiar with the use of ultrasound to diagnose tendon ruptures.

## INTRODUCTION

Musculoskeletal complaints are common in emergency medicine.^1^ On initial evaluation in the emergency department (ED), clinical assessment of musculoskeletal problems is comprised of history, physical examination, and plain radiography (which is limited in ability to evaluate soft tissue injuries). An alternative to plain radiography includes magnetic resonance imaging (MRI), a costly, time-consuming, and much less readily available modality. MRI provides excellent anatomic detail but only provides static images, and can be challenging to obtain from the ED. Ultrasound has been used to evaluate musculoskeletal structures and offers bedside static and dynamic imaging of musculoskeletal structures that is rapid and inexpensive.^2^ The American College of Emergency Physicians (ACEP) Emergency Ultrasound Guidelines address the use of ultrasound for a wide range of indications, both diagnostic and therapeutic, including musculoskeletal injuries.^3^ This article discusses a case in which point-of-care ultrasound (POCUS) led to the diagnosis of bilateral patellar tendon rupture following minor trauma.

## CASE REPORT

**Image 1:**
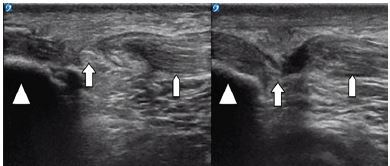
Ultrasound: the left and right correspond to the patient’s left and right patellar tendons. Orientation marker is directed toward the patient’s head and the probe is placed just at the inferior edge of the patella in a sagittal plane. 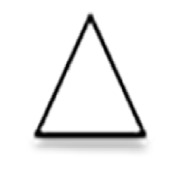
 Hyperchoic cortex of the patella with resulting anechoic shadow behind 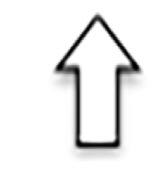
 Disruption of the patellar tendon fibers on the left and right patellar tendons 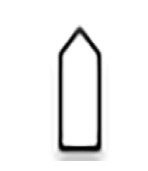
 Normal fibrillar appearance of patellar tendons on the patient’s left and right knees

A 38-year-old man presented to the ED complaining of bilateral knee pain and inability to ambulate after hopping off a ledge that was a foot or two off the ground. Upon landing, he immediately felt knee pain bilaterally, and was unable to walk. In the ED he denied any prior episodes of knee injury or pain, or any past medical history or medications – specifically no connective tissue disease or steroid use. On examination, he had swelling and a palpable defect inferior to the patella bilaterally. His neurovascular exam was normal; however, he was unable to actively extend either of his legs at the knees or lift his lower legs off the stretcher. POCUS using a high-frequency linear probe (15-8 megahertz, Sonosite XPorte, Bothell, WA) revealed bilateral patellar tendon ruptures with proximal retraction of the patella (Image 1; Video 1 and 2). Both videos are oriented in the longitudinal plane images, as the ones captured in the transverse plane did not add diagnostic value in this case and the defects were well visualized in the longitudinal plane.

**Image 2:**
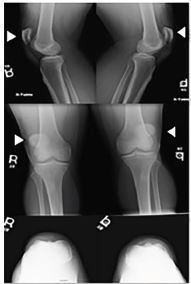
Radiograph of bilateral knees demonstrating patella alta (arrows).

He was then evaluated by orthopedics and noted to have visible deformity of bilateral knees suggestive of bilateral patella alta. He was able to contract his quadriceps bilaterally but unable to perform straight leg raise bilaterally. He was otherwise neurovascularly intact. Plain radiographs were significant for bilateral patella alta (Image 2).

The patient was subsequently admitted to the orthopedics service and underwent successful operative repair of bilateral ruptured patellar tendons.

## DISCUSSION

The extensor mechanism of the knee is essential to the ability to walk. This extensor mechanism can be disrupted by a rupture of the patellar or quadriceps tendon, or by a fracture of the patella itself with rupture of the capsule. In patients under the age of 40, patellar tendon rupture is most common in athletic adults and most often unilateral.^4^ Bilateral patellar tendon ruptures are exceedingly rare, especially in patients without underlying disease. Most cases of patellar tendon ruptures occur in patients with a predisposition towards tendinopathy due to diabetes, renal failure, lupus, rheumatoid arthritis, or corticosteroids.^4^.^5^.^6^

In the urgent setting, the use of ultrasound can expedite diagnosis and mobilize consultants to facilitate excellent patient care. Ultrasound is ideal due to its portability, low cost, and lack of ionizing radiation. Extremity and tendon injuries are especially amenable to ultrasound due to the superficial location of these structures.^7^,^8^ To ultrasound the extensor tendons of the knee, a high-frequency linear probe is used to scan in longitudinal and transverse planes. In a patient with a unilateral injury, the asymptomatic extremity may be examined for comparison.

Tendons visualized by ultrasound have a bright fibrillar structure and normally exhibit the property of anisotropy, which means their echogenicity varies depending on the angle of the ultrasound beam in relation to the tendon. Tendinous fibers will appear more echogenic or brighter if the ultrasound is perpendicular but will become less echogenic as the angle decreases. This is important in the evaluation of tendons because a ruptured tendon will appear hypoechoic or in some cases anechoic. In a ruptured tendon, hypoechoic or anechoic areas and discontinuity of the fibrillar lines with frayed appearance may be appreciated. Hypoechoic surrounding edema and hematoma may also be seen. Dynamic ultrasound of tendons during contraction of the attached muscle may aid in demonstrating partial and complete rupture by magnifying the defect.^9^

**CPC-EM Capsule**

What do we already know about this clinical entity?

Bilateral patellar tendon rupture is rare in the absence of predisposing conditions for tendinous injury such as lupus, rheumatoid arthritis, diabetes, renal disease, or chronic steroid use.

What makes this presentation of disease reportable?

This atypical presentation of disease with minimal mechanism is unexpected in a young, healthy male patient. Immediate visualization of the tendon rupture facilitates proper specialty care.

What is the major learning point?

Systematic use of point-of-care ultrasound for musculoskeletal injuries is fast, cost-effective, and allows for dynamic assessment of the tendon mechanism.

How might this improve emergency medicine practice?

Given its low cost and ease of use, point-of-care ultrasonography for musculoskeletal injury may expedite specialty consultation and treatment, particularly in resource limited locations.

While there is a learning curve to performing this examination, the ACEP Guidelines for Point-of-Care and Clinical Ultrasound suggest emergency physicians should be able to recognize tendon rupture and laceration.^3^ According to Li et al., “Despite its benefits and widespread adoption in general medicine and other specialties, however, ultrasonography is not as well adapted as a diagnostic and research tool in orthopedic surgery.”^10^ This suggests a potential symbiotic relationship that is likely to improve patient care when experienced sonographer clinicians identify pathology in collaboration with specialist colleagues. In this case, POCUS combined with history, physical exam, and plain radiographs demonstrated bilateral patellar tendon ruptures in a patient without predisposing factors, expediting orthopedic evaluation and surgical repair.

## CONCLUSION

Extensor mechanism ruptures are high-risk events that require surgical intervention. We describe a case of a 38-year-old healthy man with bilateral patellar tendon rupture diagnosed at the bedside using point-of-care ultrasound. This case highlights the importance of POCUS for musculoskeletal indications, illustrating how physicians can collaborate in the use of ultrasound to complement other traditional musculoskeletal examination modalities to expedite the diagnosis and treatment of this orthopedic surgical urgency.

## Supplementary Information

Video 1. Right patellar tendon video demonstrating rupture.

Video 2. Left patellar tendon video demonstrating rupture.
